# Supplementary material for: Mutations in PCYT2 disrupt etherlipid biosynthesis and cause a complex hereditary spastic paraplegia
Source: Brain. 2019 Oct 22;142(11):3382–97. doi: 10.1093/brain/awz291 (PMC6821184; doi:10.1093/brain/awz291)
Supplement: awz291_Supplementary_Data [file awz291_supplementary_data.zip › awz291-Suppl_data/Supplementary_Data4.zip!/Supplementary information 3 - fibroblast lipidomics.zip!/Fibroblast lipidomics/Controle vs Patient2/Statistical plots and data/Heatmap VIP-score bigger than 1.pdf]

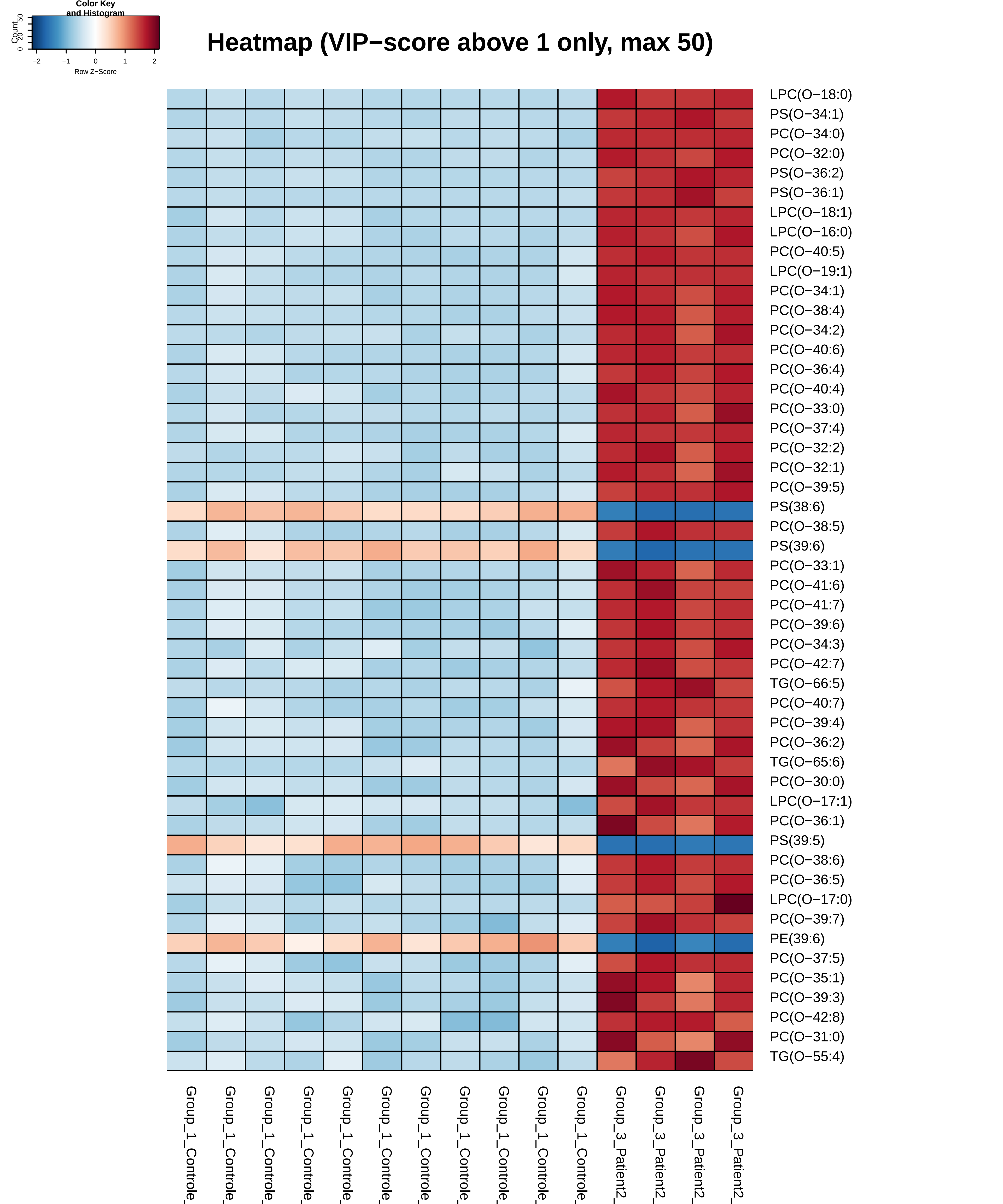
Color Key
and Histogram
Count
0 20 50
−2 −1 0 1 2
Row Z−Score
Heatmap (VIP−score above 1 only, max 50)
LPC(O−18:0)
PS(O−34:1)
PC(O−34:0)
PC(O−32:0)
PS(O−36:2)
PS(O−36:1)
LPC(O−18:1)
LPC(O−16:0)
PC(O−40:5)
LPC(O−19:1)
PC(O−34:1)
PC(O−38:4)
PC(O−34:2)
PC(O−40:6)
PC(O−36:4)
PC(O−40:4)
PC(O−33:0)
PC(O−37:4)
PC(O−32:2)
PC(O−32:1)
PC(O−39:5)
PS(38:6)
PC(O−38:5)
PS(39:6)
PC(O−33:1)
PC(O−41:6)
PC(O−41:7)
PC(O−39:6)
PC(O−34:3)
PC(O−42:7)
TG(O−66:5)
PC(O−40:7)
PC(O−39:4)
PC(O−36:2)
TG(O−65:6)
PC(O−30:0)
LPC(O−17:1)
PC(O−36:1)
PS(39:5)
PC(O−38:6)
PC(O−36:5)
LPC(O−17:0)
PC(O−39:7)
PE(39:6)
PC(O−37:5)
PC(O−35:1)
PC(O−39:3)
PC(O−42:8)
PC(O−31:0)
TG(O−55:4)
Group_1_Controle_
Group_1_Controle_
Group_1_Controle_
Group_1_Controle_
Group_1_Controle_
Group_1_Controle_
Group_1_Controle_
Group_1_Controle_
Group_1_Controle_
Group_1_Controle_
Group_1_Controle_
Group_3_Patient2_
Group_3_Patient2_
Group_3_Patient2_
Group_3_Patient2_
